# Biology and Johns Hopkins University

**Published:** 1881-05

**Authors:** 


BIOLOGY AND JOHNS HOPKINS UNIVERSITY.—We should have noticed the circulars of our great University, and the subject of biology particularly, which is now being taught so thoroughly and satisfactorily by and under the auspices of the able faculty, but procrastination interposed, and beguiled us on until the next circular should be received. A course of instruction in biology has been so arranged that young men proposing to study medicine can take a course in this department, preliminary to entering regularly upon their studies, that cannot fail to prove of great advantage to such as may avail themselves of it. There are two major and three minor courses. The major course includes chemistry and biology, while in the minor course are physics, mathematics, logic, psycology, French and German, all of which will be found of great preliminary and future advantage to the medical student. The course on biology explains the laws of life in general, as seen in unicellular and higher organisms. Mammalian anatomy and physiology are next taught, with the use of the microscope. Human and comparative anatomy come next, and are taught, together with dissection of certain mammals. Added to these, laboratory work comprises a considerable portion of the course.

Thoroughness is aimed at in all the branches taught in the University, and, when organized and in operation in all its departments, it will not be surpassed, if equalled even, by any university in the world. Not only Baltimore, but our entire country should feel proud that we have such a grand institution on this Western Continent as the Johns Hopkins University.

